# Impacts of Tree Rows on Grassland Birds and Potential Nest Predators: A Removal Experiment

**DOI:** 10.1371/journal.pone.0059151

**Published:** 2013-04-02

**Authors:** Kevin S. Ellison, Christine A. Ribic, David W. Sample, Megan J. Fawcett, John D. Dadisman

**Affiliations:** 1 Department of Forest and Wildlife Ecology, University of Wisconsin, Madison, Wisconsin, United States of America; 2 U.S. Geological Survey, Wisconsin Cooperative Wildlife Research Unit, University of Wisconsin, Madison, Wisconsin, United States of America; 3 Wisconsin Department of Natural Resources, Bureau of Science Services, Madison, Wisconsin, United States of America; University of Milan, Italy

## Abstract

Globally, grasslands and the wildlife that inhabit them are widely imperiled. Encroachment by shrubs and trees has widely impacted grasslands in the past 150 years. In North America, most grassland birds avoid nesting near woody vegetation. Because woody vegetation fragments grasslands and potential nest predator diversity and abundance is often greater along wooded edge and grassland transitions, we measured the impacts of removing rows of trees and shrubs that intersected grasslands on potential nest predators and the three most abundant grassland bird species (Henslow’s sparrow [*Ammodramus henslowii*], Eastern meadowlark [*Sturnella magna*], and bobolink [*Dolichonyx oryzivorus*]) at sites in Wisconsin, U.S.A. We monitored 3 control and 3 treatment sites, for 1 yr prior to and 3 yr after tree row removal at the treatment sites. Grassland bird densities increased (2–4 times for bobolink and Henslow’s sparrow) and nesting densities increased (all 3 species) in the removal areas compared to control areas. After removals, Henslow’s sparrows nested within ≤50 m of the treatment area, where they did not occur when tree rows were present. Most dramatically, activity by woodland-associated predators nearly ceased (nine-fold decrease for raccoon [*Procyon lotor*]) at the removals and grassland predators increased (up to 27 times activity for thirteen-lined ground squirrel [*Ictidomys tridecemlineatus*]). Nest success did not increase, likely reflecting the increase in grassland predators. However, more nests were attempted by all 3 species (175 versus 116) and the number of successful nests for bobolinks and Henslow’s sparrows increased. Because of gains in habitat, increased use by birds, greater production of young, and the effective removal of woodland-associated predators, tree row removal, where appropriate based on the predator community, can be a beneficial management action for conserving grassland birds and improving fragmented and degraded grassland ecosystems.

## Introduction

Globally, grassland habitats and the animals that depend upon them are widely imperiled by the degradation and fragmentation associated with agricultural and energy development, succession, and urbanization [1]. Beyond the direct loss of grassland habitat through conversion to cropland, the loss of historical forces such as fire and free-ranging large grazers (behaviorally influenced by predators [2]) has altered the extent and quality of habitats at the landscape level [3], [4]. Additionally, abandoned agricultural lands have undergone habitat state-transitions, primarily through vegetative succession to shrublands or woodlands [3], [5], as fire and grazers are not allowed to function fully (under or over grazing) to abate encroachment by trees and shrubs [4]. Thus, in the past 150 years, woody vegetation has become more widespread in grasslands and savannas of North and South America, Africa, Australia, and southeast Asia [6], [7]. This encroachment can fundamentally change grasslands, jeopardizing biodiversity and threatening the sustainability of pastoral, subsistence, and commercial livestock grazing [8], [9]. Such changes may adversely impact up to 20% of the world’s population [10].

In North America, populations of 55% of 42 grassland bird species are declining and are among the fastest and most consistently (since 1966) declining species [11], [12], [13]. This is significant as grassland birds often serve as indicators of ecosystem quality [14]. The leading threats to North American grassland birds are thought to be habitat loss and degradation, including habitat fragmentation and the continued intensification of agriculture [15]. However, few studies have directly examined the impact of grassland fragmentation by woody vegetation (review by [16]), particularly with respect to impacts on the activity of potential nest predators (e.g., increased predation), especially species associated with and/or subsidized by woody vegetation. The problem of fragmentation and intrusion by woodland-associated predators has grown among Midwestern grasslands in the past 40–60 yr, as the fencing and subdivision of property has facilitated the encroachment of woody vegetation predominately in the form of linear tree rows between agricultural fields (e.g., crops, forages, former crop fields planted into grasses through the USDA’s Conservation Reserve Program, known as the CRP). To measure the responses of grassland birds and potential nest predators to the removal of woody vegetation in tree rows that intersected grassland fields, we conducted an ecological removal experiment.

Although most obligate grassland bird species will readily use shrubs or trees as perches when available, their nesting behavior and productivity is negatively impacted by woody vegetation at patch and landscape scales [16], [17], [18], [19]. In agricultural areas, woody vegetation, particularly in linear configurations, as found along fencerows with volunteer trees and shrubs and planted windbreaks adjacent to grassland habitats (e.g., pastures, CRP fields), can influence grassland birds by altering predator densities and activity along edges between habitats [20], [21], [22], [23], [24], [25]. Also, in the wooded-grassland matrix common in much of Midwestern and Eastern North America, potential nest predators typical of woodland-habitats will use tree rows as travel corridors along grasslands [25], [26], [27]. The use of such corridors by potential predators is significant, as predation of nests [26], [27], [28] and fledged young [29], [30], [31], [32], [33] is a primary determinant of recruitment among birds [34], [35]. The identification of features that affect the probability of predation can be useful for improving efforts aimed at managing avian populations [36], [37].

Patterns of bird settlement during migration and habitat use may also be affected by woody habitat. Studies have shown that grassland birds avoided nesting within 50 m or less of wooded edges [17], [22], [24], [38]. Such behavior can effectively reduce the number of patches suitable for some grassland birds [22]. Whether the mechanism for this avoidance reflects a behavioral response to woody vegetation or predators directly [39], the negative association between obligate grassland bird abundance and woody vegetation, points to the potential for benefits associated with the removal of wooded edges for bird conservation –more grassland habitat would effectively be available for grassland birds.

To test these hypotheses, we conducted an ecological removal experiment designed to measure the responses of grassland birds and potential nest predators to the removal of woody vegetation in tree rows that intersected grassland fields. We predicted that more grassland birds would use the habitat adjacent to fence rows after the woody vegetation was removed. Specifically, we expected more bird territories within 50 m of fence rows after the removal of woody vegetation. At each treated site, we expected more nesting attempts with greater daily nest survival and productivity (number of nests fledging at least 1 young) overall, particularly within 50–100 m of the areas where trees were removed. We also expected the increase in nest daily survival rate to be associated with a decrease in the presence of woodland-associated predators.

## Methods

### Study Sites

**Figure 1:**
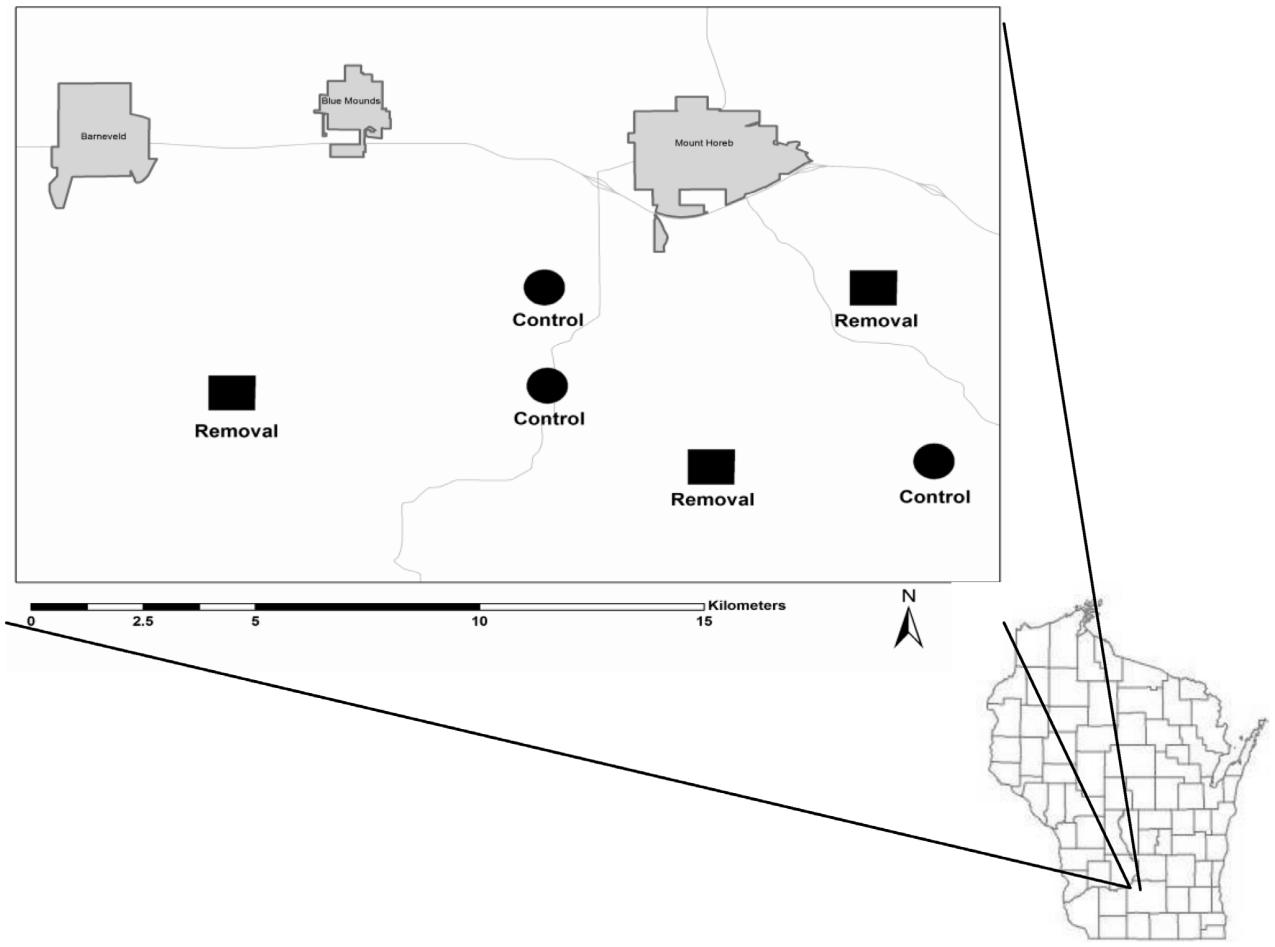
Study site locations in southwestern Wisconsin. Squares and circles indicate sites used for tree row removals and controls, respectively. Shaded polygons depict developed areas, labeled by town name. Lines represent major highways.

We conducted our study on private lands in southwestern Wisconsin, May–July 2005–2008 (Fig. 1). The study area was located in one of the most important landscapes in Wisconsin for grassland bird conservation [40], [41] and with high enrollment (15.6% of lands within 1 km of our sites) in the CRP [42]. Land enrollment in the CRP ranges from 1.6% across the state of Wisconsin, to 10.1% for the Southwestern Grassland and Streams Conservation Area.

Area land use is primarily agricultural, with relatively few acres of row crops (corn [*Zea mays*] and soybeans [*Glycine max*]) compared to many other agricultural areas of Wisconsin. This area also contains a concentration of scattered, small (1–28 ha) native grassland prairies remain on rocky soils and hilltops too steep to plow [39]. In 2008, land cover was estimated within a 1-km radius of each site and categorized as: cropland 41% (range 31–52%); pasture and grass 25% (range 14–34%); woodland 18% (range 11–25%); and savanna/shrubland 9% (range 6–13%).

We randomly selected sites with woody vegetation along fencerows, hereafter referred to as tree rows, that intersected CRP fields (see Figs. S1 and S2 for site dispersion of these characters) of cool-season European grasses, primarily smooth brome (*Bromus inermis*), other grasses, and a wide variety of forbs. Tree row vegetation at the sites was between 10–60 years old and consisted of trees (mostly black cherry [*Prunus serotina*] and box elder [*Acer negundo*]) up to 18 m tall and an understory of shrubs (e.g., prickly ash [*Zanthoxylum americanum*] and grey dogwood [*Cornus racemosa*]). Most of the CRP fields in the study area had been enrolled continuously for 15 or more years. Site sizes (grassland fields on both sides of an intersecting tree row) ranged from 35–153 ha (mean = 72 ha, SD = 44, N = 6) (see Figs. S1 and S2).

We defined each study site area at the beginning of the study (Spring 2005), as the contiguous area planted to cool season grasses (CRP), bounded by cropland, pasture, hayfields, developments (residential/farm), and woodlands (including wooded strips >20 m wide [43]) (see Figs. S1 and S2). Internal features such as wooded strips <20 m wide (including the focal tree-row), tree clumps, lone trees, county roads, and grassy farm lanes that were bordered by CRP on both sides were not considered barriers, and were included in the total calculated area per study site (see Figs. S1 and S2).

### Study Design

We randomly assigned three sites for tree row removal (treatment) and three sites for controls. Each of three treatment sites had one season of pre-removal data and then removals took place the following fall/winter (between October and February). This allowed each treatment site to have a baseline. Control and treatment sites were paired on basis of field size. Two site pairs were initiated in 2005; the third site pair was initiated in 2006 due to funding limitations.

To reduce any potential effects of a gradient in bird and/or predator response to the removals, we used disjunct areas for analysis as “edge” and “interior” (see File S1 and Figs. S1 and S2). Specifically, we used <50 m of the focal tree row (the primary tree row that separated two fields of grass) as a definition of edge and >100 m from any tree row as interior, as edge effects for grassland birds are typically found within 50 m of woody edges [17], [18].

### Woody Vegetation Removal

On treatment sites, the focal tree row (and other woody vegetation, such as clusters of trees; see Fig. S2) was removed following a first year of control data with the all woody vegetation unaltered. Two methods for removal were applied: 6–8 people with chainsaws and 2 feller bunchers; these took 30 d and 7 d, respectively, to clear tree rows. Method-based costs were $25/m for chainsaw crews and $10/m for feller bunchers, respectively (feller buncher costs were reduced by scheduling work into a larger series of projects in the region). Understory vegetation was forestry-mowed and brush was aggregated and burned or removed for firewood. Remaining stumps and stems were treated with herbicide (see File S1) to prevent re-growth. Fencing was removed, with only widely-spaced (approximately 50 m) 4-cm wide posts left to demarcate field boundaries for land owners.

### Avian Abundance

We used spot-mapping of breeding territories as represented by the numbers of singing males [44] to measure bird density. Spot-map surveys were conducted along transects 100 m apart and parallel to the focal tree rows (Figs. S1 and S2). Each site was surveyed a minimum of eight times, along the same transects, between 15-May and 4-July. Surveys were conducted between 0500 and 1000 CDT, in dry conditions (no rain or fog) and wind velocities less than 15 kph. To minimize observer bias, two observers alternated survey dates at individual sites. One observer walked along each transect slowly (approximately 1 kph) recording location and species of all singing males. Contemporaneous counter-singing males were noted along with bird movements to minimize probability of double-counting. Birds from adjacent fields using study site boundaries for singing perches, mobbing red-winged blackbirds (*Agelaius phoeniceus*), mobbing bobolinks (*Dolichonyx oryzivorus*), fledglings, birds flying over the transect, and brown-headed cowbirds (*Molothrus ater*) were noted but were not included in any analysis.

### Avian Productivity

Nest searching and monitoring were conducted from 15 May to 30 July of each year. Each field was systematically searched every 10–14 d during the breeding season between 0600 and 1000 h CDT. Nest search areas did not include internal site features or edge woody areas and thus were slightly smaller than the spot map areas (see Figs. S1 and S2). We monitored all nests of all bird species that we located.

We monitored nests by either visually checking nest contents every 3–4 d or by viewing nests via a remote video-recording system (see camera deployment below). Use of data collected from nests monitored with both methods is supported as a meta-analysis did not find clear evidence that remotely monitoring nests with cameras affects nest survival [45]. Overall, we monitored 70.5% of nests remotely; bobolink (59.8% of nests); Eastern meadowlark (77% of nests); and Henslow’s sparrow (90% of nests). During each visit we recorded the number of eggs and young (host species and cowbird), condition of nest and contents, and the presence of adults at or near the nest. We recorded nests as successful if at least one young of the parental species fledged.

### Identification of Nest Predators

We deployed video systems that included miniature cameras with infra-red (950 nm) light-emitting diodes (LEDs) [46] at randomly selected grassland bird nests to determine nest fates and to identify sources of nest failure. Effort was taken to place cameras equally at nests near (<100 m) and far (>100 m) from the focal tree row. We followed the recommendations of [45] when deploying cameras: we distributed cameras within and among fields to ensure that no clustering of cameras occurred and we delayed camera placement at nests until the egg-laying stage to reduce the chance of abandonment. We prioritized setting up cameras as early in the incubation period as possible to obtain a similar number of observation days during incubation and nestling stages.

We reviewed video footage to determine each nest’s fate. We identified nest predators from the recordings with the help of researchers from the Wisconsin Department of Natural Resources. Species depredating nests were classified to the lowest taxonomic level possible (i.e., class, genus, or species).

### Activity of Potential Predators

To determine the presence and activity rates of potential and known nest predators, we used sand track stations following the protocol of [24]. We used 4 sets of 4 stations at each site, with 2 sets far (>100 m) from the focal tree row and 2 sets within the focal tree row (Figs. S1 and S2); this ensured sampling an equal amount of edge and interior on an individual site. Within a set, track stations were placed 30 m apart from each other, parallel to the focal tree row (Figs. S1 and S2).

Each station was a 1-m^2^ circle of fine sand (approximately 18 L) mixed with 250 ml of mineral oil. Mineral oil was used to improve track clarity and is non-toxic and odorless so it would not attract animals to the stations. A white, 2.5 cm diameter, unscented Plaster of Paris disk (Pocatello Supply Depot, Pocatello, ID) was placed in the center of the track station as a novel, visual stimulus to facilitate sampling predators that passed nearby by stimulating investigation that would leave tracks within the station.

We checked track stations every other day to allow time for predator response and to minimize disturbance by weather. We recorded date, time, species, track measurements and weather information for each sampling period. Since tracks are unreliable for distinguishing individuals, we considered each station independently [21] and counted multiple registries by the same species as a single visit for that species. For our analyses, we used the activity data for all species confirmed as predators with video at nests and for which >100 track detections were recorded.

To better identify the snake species that used the sites, we used a cover board survey [47], of CRP fields adjacent to the study areas. Adjacent fields (Figs. S1 and S2) were used to avoid influencing our primary study sites as cover boards may alter habitats available to small rodents, snakes, and/or indirectly, birds. The adjacent fields were similar in vegetation structure and composition, as well as time since enrollment in the CRP. Cover boards were 121×91 cm, sanded, 1 cm-thick pieces of exterior-grade pine plywood. The boards were placed in grids of four, 50 m-spaced rows of five boards each (20 boards/site), placed parallel to a tree row in an adjacent field, or when space permitted, in the same field outside of the bird/nest sampling areas (see Figs. S1 and S2). Boards within a row were 15 m apart. Arrays were checked for snakes every other day. Because not all snakes were large enough to ingest eggs, we categorized individuals as potential predators, or not, based on their size. Head size relative to potential prey items of eggs or young was evaluated sensu [48], with reference to [49] (40 cm snakes consumed quail eggs) and [50] (snake and prey size allometry). Body length of 20 cm or greater was determined as the criterion for ability to depredate nests of the study species.

The protocol for this study was approved by the Animal Care and Use Committee of the University of Wisconsin-Madison (Permit Number: A-1210).

### Data Analysis

We focused data analysis on the three obligate grassland bird species that occurred on all the sites (bobolink, Eastern meadowlark (*Sturnella magna*), and Henslow’s sparrow (*Ammodramus henslowii*)); these species made up 75% of the grassland birds detected during spot map surveys and the nests of these species accounted for 91% of all nests found. These species also require grasslands for all parts of their breeding cycle and are “Species of Greatest Conservation Need” in Wisconsin [41]. Henslow’s sparrow is listed as state-threatened in Wisconsin [40].

To analyze the activity of the most common nest predator species identified using nest video recordings, we used data from species or groups of species (e.g., small rodents) whose tracks were recorded >100 times; these were the raccoon (*Procyon lotor*), white-tailed deer (*Odocoileus virginianus*), coyote (*Canis latrans*), striped skunk (*Mephitis mephitis*), thirteen-lined ground squirrel (*Ictidomys tridecemlineatus*), small rodents (pooled species), and snakes (pooled species).

### Analysis 1: Edge and Interior

We analyzed the edge and interior areas (Figs. S1 and S2) separately because we did not know how far into the field the effects of the removal might be seen. Assuming no effects in the interior and comparing the edge to the interior data would be erroneous if both areas were affected by the treatment. Therefore, we compared edges and interiors between paired control and treatment sites. Study site, focal tree row, spot map, and nest search areas were calculated from geo-referenced aerial imagery digital orthophotos [51] using ArcGIS version 9.3 software [52]. The focal tree row area was subtracted from the edge spot map and nest search areas for pre-treatment years and at control sites.

For birds, species density was based on the spot maps. Because error in singing male locations could be as great as 25 m, we considered centroids ≤75 m of the focal tree row as males using the edge and centroids ≥100 m from the focal tree row as males using the interior (Figs. S1 and S2). We calculated density (males/ha) for the areas for edges and interiors by dividing the number of centroids by area surveyed.

Avian species density (males/ha) and nest predator activity rates (visits/station/day) were estimated on a per site basis. We used standardized visitation rates (visits/station/day) as an index to predator species activity. Nest density was calculated similarly, with two caveats. First, because nest location was known more precisely than male locations (for species density), nests ≤50 m of the focal tree row were considered to occur in edge habitat along the focal tree row (or, at removals, along the edge where the tree row once was) while those ≥100 m from the focal tree row were considered in the field interior (Figs. S1 and S2). Second, because it is unlikely that we accounted for all nests initiated, we refer to the number of nests divided by the area searched as “apparent” nest density (nests/ha). We were unable to estimate the number of nests initiated and not found (sensu [53]) because we were unable to assign nest age to a majority of nests found (38% of 340 nests were of known age).

To control for any potential effects due to field size, we paired the treatment and control sites by size. Paired plots followed over time are well-suited for standard analysis of covariance where the objective is to use a strong explanatory relationship to reduce the error term relative to what it would be if the data were analyzed with a standard control/treatment linear model [54]. Thus, the paired-plot Model 1 was
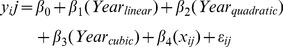
(1)where:

*y_ij_* = response variable observed on treatment plot;

*x_ij_* = response variable observed on control plot (the covariate being tested);

i = 1, 2, 3, … indexes site pairs;

j = 0, 1, 2, 3indexes year relative to when the plot’s series began;

*Year* = 0 pre-treatment;

1. first year post-treatment;
2. second year post-treatment;
3. third year post-treatment;
4. Year*_linear_,* Year*_quadratic_*, and Year*_cubic_* = linear, quadratic, and cubic trends in treatment response over time; and
5. *ε_ij_* = error term with Normal (0, σ^2^) distribution.

The decomposition of treatment effect into linear, quadratic, and cubic components allowed us to characterize the treatment response. The polynomial characterization of the response is useful for understanding the nature of the effect – increasing over time, a perturbation in the mean that is sustained over time, or a perturbation in the mean that decays over time to the original state. When possible, we simplified the model by removing non-significant (P>0.10) trend components.

When the covariate relationship is not strong (e.g., P>0.10), the error term is not reduced as expected, resulting in a test of treatment effect that is not as powerful as that provided by the standard control/treatment linear model [54]. This is a serious concern with small sample sizes, such as we have here. Therefore, for each response variable, the strength of the covariate relationship in paired-plot Model 1 was evaluated. When the P-value for the covariate was >0.10, indicating the lack of a strong explanatory relationship, we replaced the paired-plot model with the standard control/treatment linear model. In this model, time trends in the treatment were compared against a baseline of the control sites and the pre-treatment values. Time trends were considered significant at P<0.05 and indicative of an effect at 0.05<P<0.10.

### Analysis 2: Nesting Success

Due to a limited number of nests by species by site, particularly for the control sites and the pre-removal year for the treatment sites, analyses were done with nests pooled across sites (i.e., by Control and Tree row removal). We used the logistic exposure method [55] to determine if nesting success differed among years for the control sites. We then analyzed nesting success in relation to the treatment (Control, Pre-removal, Year 1, Year 2, and Year 3 post removal), distance to the focal and nearest woody edge(s), and interactions. We used AICc to rank the models [56] and calculated AICc weights to assess the importance of the different models [56]. Analyses were done using R [57]. To explore any impact of nest cameras, we included a camera-effect in our nest success models and that variable was not significant.

We used daily survival rate (DSR) and nesting period for each species to calculate the probability of fledging young per nest attempt. We used a 23-d nesting period (incubation and nestling period combined) for bobolink [58], 21 d for Henslow’s sparrow [59], and 24 d for Eastern meadowlark [25].

### Analysis 3: Nest Predators

Nest predators identified from the video recordings at nests were categorized into grassland-associated mammals or birds, woodland-associated mammals, and snakes. Classification of predators into groups followed Ribic et al. [25]. Due to low numbers of predation events by site, data were combined into Control and Tree row removal categories. We calculated the proportions of predation by category and we tested whether the proportions of the nests depredated by the different predator categories over time was the same between the control and tree row removal categories using a Chi-square test and standardized residual analysis [60]. To avoid reliance on asymptotic results, we used a Monte-Carlo simulation approach [61]. Significance was assessed at alpha = 0.05.

## Results

### Density

Bobolink, Eastern meadowlark, and Henslow’s sparrow were the common grassland species found on all control and tree row removal sites, accounting for 75% of the grassland birds detected. The covariate relationship between the paired sites was not significant for any of the three species (P>0.25, all tests). Therefore, we used the treatment/control linear model in further analyses. Within the control sites, there were no significant year-effects on density, regardless of species (P>0.25, all tests). Densities on the control sites were homogenous across years and were best modeled using a single control parameter (see Table 1).

**Table 1:** Mean male density (males/ha) and standard errors (in parentheses) for obligate grassland birds in tree row edge and grassland interior habitats at the control and treatment sites.

	Bobolink		Eastern meadowlark		Henslow's sparrow	
	Edge (SE)	Interior (SE)	Edge (SE)	Interior (SE)	Edge (SE)	Interior (SE)
Control	0.265 (0.043)	0.641(0.121)	0.321 (0.094)	0.299 (0.024)	0.066 (0.043)	0.223 (0.078)
Pre-removal	0.449 (0.162)	0.585 (0.324)	0.343 (0.166)	0.120 (0.029)	0.015 (0.015)	0.206 (0.046)
Year 1	0.664 (0.178)	0.751 (0.337)	0.254 (0.128)	0.147 (0.024)	0.177 (0.177)	0.172 (0.090)
Year 2	0.833 (0.113)	0.938 (0.444)	0.332 (0.011)	0.154 (0.044)	0.249 (0.125)	0.295 (0.133)
Year 3	0.722 (0.323)	1.143 (0.755)	0.285 (0.063)	0.174 (0.084)	0.242 (0.068)	0.298 (0.018)

Densities for bobolinks and Henslow’s sparrows along tree row edge increased after removal (linear trends: bobolink: F = 13.54, df = 1, 18, P = 0.002; Henslow’s Sparrow: F = 5.19, df = 1, 18, P = 0.035). Following removals, bobolink and Henslow’s sparrow densities at edges were 1.5–4 and 2–4 times greater than in pre-treatment seasons and at control sites, respectively (Table 1). Eastern meadowlark densities did not change at edges after removal (F = 0.04, df = 1, 18, P = 0.83) (Table 1).

Within field interiors, no species showed a density response after removal (bobolink: F = 2.26, df = 1, 18, P = 0.15; Eastern meadowlark: F = 0.80, df = 1, 17, P = 0.39; Henslow’s sparrow: F = 0.49, df = 1, 18, P = 0.49) (Table 1).

### Apparent Nest Density

For all three species, apparent nest density increased at edges after removal (linear trend: bobolink: F = 15.77, df = 1, 6, P = 0.008; Eastern meadowlark: F = 6.68, df = 1,14, P = 0.022; Henslow’s sparrow: F = 7.22, df = 1, 18, P = 0.015) (Table 2).

**Table 2:** Mean apparent nest density (number/ha) and standard errors (in parentheses) for obligate grassland birds in tree row edge and grassland interior habitats on the control and removal sites.

	Bobolink		Eastern meadowlark		Henslow's sparrow	
	Edge (SE)	Interior (SE)	Edge (SE)	Interior (SE)	Edge (SE)	Interior (SE)
Control	0.149 (0.071)	0.333 (0.097)	0.569 (0.143)	0.630 (0.148)	0–	0.019 (0.012)
Pre-removal	0.129 (0.065)	0.249 (0.145)	0.148 (0.148)	0.132 (0.093)	0–	0.051 (0.051)
Year 1	0.301 (0.108)	0.254 (0.181)	0.694 (0.142)	0.116 (0.036)	0.089 (0.089)	0.069 (0.037)
Year 2	0.510 (0.190)	0.379 (0.126)	0.631 (0.200)	0.446 (0.109)	0.199 (0.121)	0.111 (0.073)
Year 3	0.581 (0.046)	0.601 (0.476)	0.937 (0.310)	0.382 (0.305)	0.104 (0.104)	0.070 (0.007)

Apparent nest density for bobolinks and Eastern meadowlark on the treatment sites did not increase in the interiors after removal (bobolink: F = 1.1, df = 1, 18; P = 0.29; Eastern meadowlark: F = 1.41, df = 1, 17, P = 0.25) (Table 2). Henslow’s sparrow had a small increase at interiors after removal (linear trend: F = 3.2, df = 1, 18, P = 0.09) (Table 2). Henslow’s sparrow was only found nesting at interiors on pre-removal and control sites.

**Figure 2:**
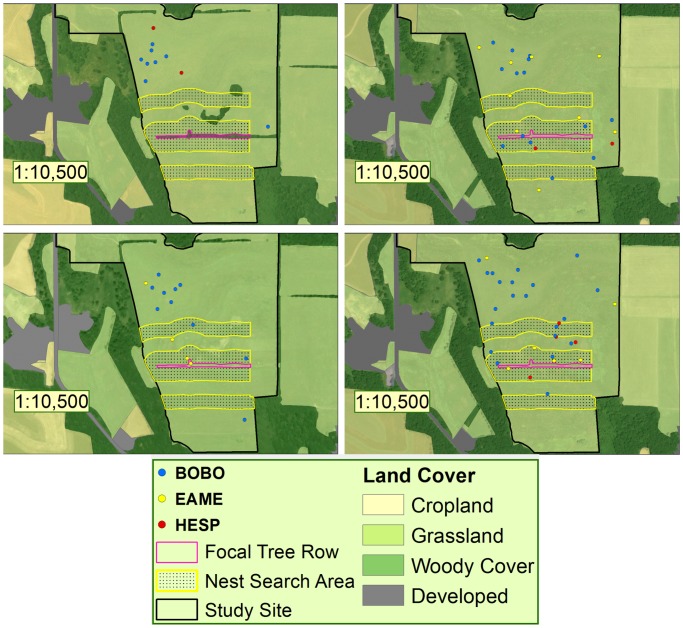
Annual maps of nest dispersion. Nests of the 3 focal species relative to the focal tree row, edge, and interior habitat at a tree row removal site in southwest Wisconsin. More nests occurred in the tree row edge area following the removal of woody vegetation. Dots refer to nest locations by species, with: blue for bobolink, yellow for Eastern meadowlark, and red for Henslow’s sparrow.

In summary, following removals, nesting by all species increased in edges (see Fig. 2). Indeed, meadowlark and bobolink nests were found within 1 m of locations where trees were present 4–5 months prior (Fig. 2). Henslow’s sparrow did not nest in edges until after the tree row removal.

### Nest Success

DSR did not differ among the years for nests on the control sites for any of the species (P>0.10, all models). DSR also did not differ among the control sites and the pre-removal year for the treatment sites for bobolink and Eastern meadowlark (P>0.10, all models); data were insufficient for testing DSR for Henslow’s sparrow. Probability of fledging at least one young on the control sites and before treatment did not differ among species (Table 3).

**Table 3:** The probabilities (Daily Survival Rates) for nests fledging ≥1 young, listed by species pooled at the whole field level by treatment.

	Nests	Probability of fledging 1 young (95% C.I.)	Successful nests
**Control & Pre-removal**			
Bobolink	40	0.3588 (0.1833, 0.5404)	25
Eastern meadowlark	69	0.3726 (0.2574, 0.4882)	32
Henslow’s sparrow	7	0.2734 (0.0456, 0.5878)	2
Pooled	116	0.36186 (0.06697, 0.41567)	59
**Removals**			
Bobolink	67	0.31820 (0.11515, 0.50788)	35
Eastern meadowlark	84	0.185396 (0.07466, 0.33688)	28
Henslow’s sparrow	24	0.193496 (0.02901, 0.49330)	11
Pooled	175	0.237354 (0.08390, 0.42380)	74

Because control and treatment sites were paired, the areas and time-periods are comparable. However, due to the low number of nests near tree rows, data were pooled for the control sites and pre-removal years.

**Figure 3:**
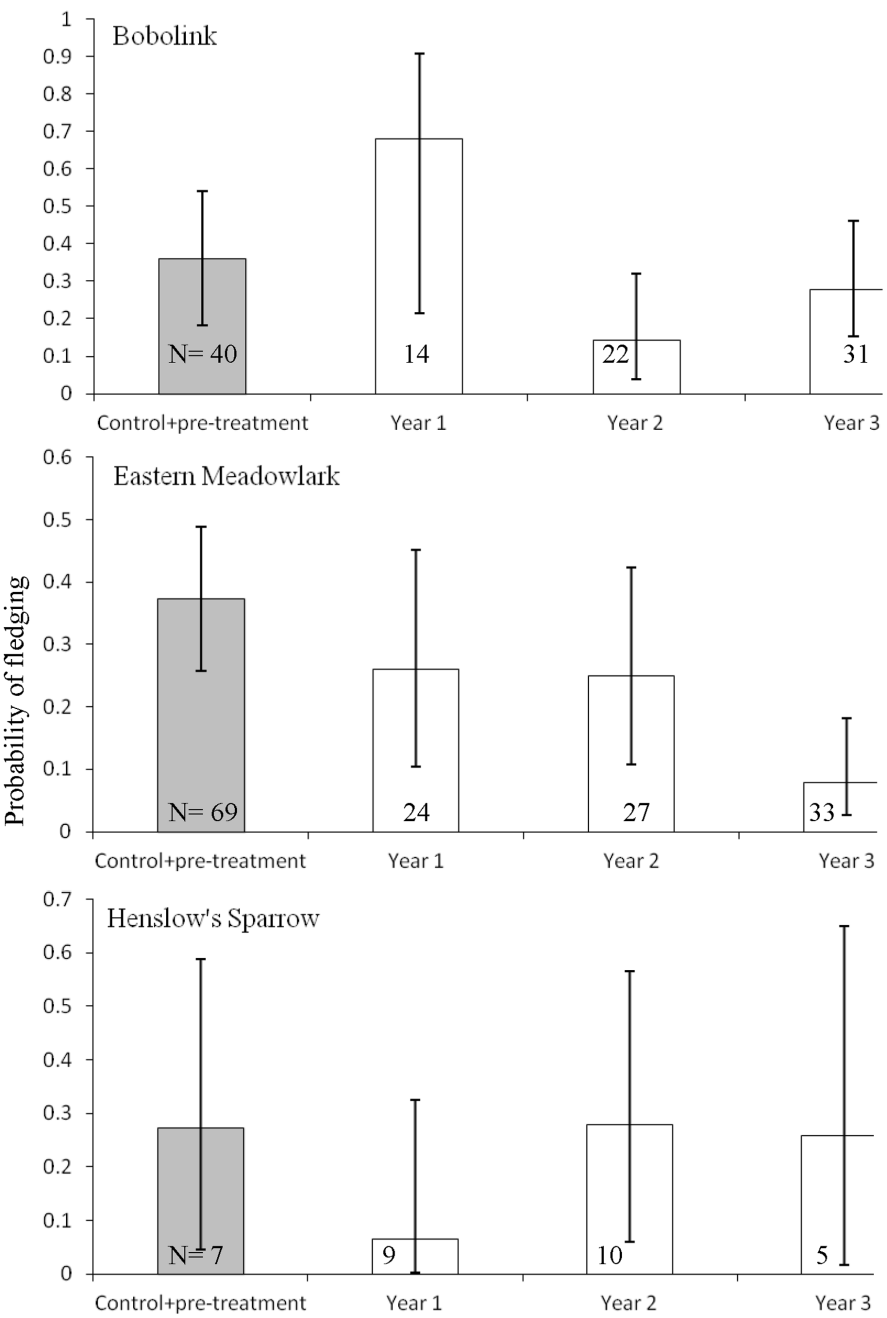
Probability of fledging ≥1 young by species and treatment. Because control and tree row removal sites were paired, the areas and time-periods are comparable. Due to the low number of nests near tree rows (egdes), data were pooled for the control sites and pre-treatment years.

Nest DSR for bobolink and Eastern meadowlark was affected by the removal of the tree row and distance to the nearest edge (Tables 4 and 5). Specifically, DSR for bobolink nests increased the first year after tree row removal and then decreased the second year after removal compared to the control sites (Fig. 3). DSR for Eastern meadowlark nests was not different from the control sites the first two years after tree row removal but then decreased the third year after removal (Fig. 3). For both species DSR increased the farther the nest was from any edge. A bobolink nest 30 m from an edge had a probability of fledging of 0.633 while a nest 120 m away from an edge had a probability of fledging of 0.818. The probability for an Eastern meadowlark nest was 0.230 and 0.427 for distances of 30 and 100 m, respectively. Henslow’s sparrow nest DSR did not differ between the treatment and control sites (Fig. 3). However, Henslow’s sparrow did not nest at edges until after removals.

**Table 4:** AIC results for nest success on control and tree row removal sites.

Species	Model	n	K	AICc	deltaAIC	weight
Bobolink	Distance to nearest edge+removal	911	6	313.72	0.00	0.625
	Distance to focal tree row+removal	911	6	317.28	3.56	0.106
	Distance to nearest edge	911	3	317.92	4.20	0.077
	Distance to nearest edge* removal	911	9	318.06	4.34	0.071
	Distance to focal tree row	911	3	319.02	5.30	0.044
	Removal	911	5	319.17	5.45	0.041
	Constant	911	2	320.20	6.48	0.024
	Distance to focal tree row* removal	911	9	321.78	8.06	0.011
Eastern meadowlark	Distance to nearest edge+removal	1703	6	602.18	0.00	0.635
	Distance to focal tree row+removal	1703	6	604.89	2.71	0.164
	Removal	1703	5	606.22	4.04	0.084
	Distance to nearest edge* removal	1703	9	606.51	4.33	0.073
	Distance to nearest edge	1703	3	609.41	7.23	0.017
	Distance to focal tree row	1703	3	609.93	7.75	0.013
	Distance to focal tree row* removal	1703	9	610.59	8.41	0.009
	Constant	1703	2	612.41	10.23	0.004
Henslow's sparrow	Constant	267	2	121.25	0.00	0.518
	Distance to focal tree row	267	3	123.28	2.03	0.188
	Distance to nearest edge	267	3	123.29	2.03	0.188
	Removal	267	5	125.64	4.38	0.058
	Distance to focal tree row+removal	267	6	127.61	6.35	0.022
	Distance to nearest edge+removal	267	6	127.69	6.44	0.021
	Distance to nearest edge* removal	267	9	131.33	10.07	0.003
	Distance to focal tree row* removal	267	9	131.44	10.19	0.003

**Table 5:** Coefficients for the minimum AICc models for bobolink and Eastern meadowlark from Table 4.

Species	Term	Coefficient	Estimate Std. Error	z value	P
Bobolink	Intercept	2.36	0.365	6.481	<0.001
	Distance to nearest edge	0.009	0.004	2.621	0.009
	Removal Year 1	1.264	0.771	1.639	0.101
	Removal Year 2	−0.752	0.390	−1.928	0.054
	Removal Year 3	−0.278	0.373	−0.746	0.455
Eastern meadowlark	Intercept	2.820	0.215	13.089	<0.001
	Distance to nearest edge	0.006	0.003	2.375	0.018
	Removal Year 1	−0.242	0.323	−0.749	0.454
	Removal Year 2	−0.476	0.305	−1.557	0.119
	Removal Year 3	−1.005	0.270	−3.719	<0.001

Importantly, the nest data provide a whole field assessment, with the greatest numbers of nesting attempts and successful nests at the removal sites, overall, for Henslow’s sparrow and bobolink (Table 3). The observed and expected (based on DSR) number of nests fledged on removals was greater than at controls and pre-removal for bobolink (35 and 21.2 versus 25 and 14.4, respectively) and Henslow’s sparrow (11 and 4.7 versus 2 and 1.9, respectively). Despite a decrease in DSR among Eastern meadowlark nests, the number of successful nests at removals was similar to that at controls and pre-removal (32 versus 28).

### Identification of Nest Predators

We recorded 30 nest predation events at control sites; of these, 80% were grassland-associated mammals, 10% were woodland-associated mammals, 7% were snakes, and 3% were grassland-associated birds. We considered the grassland-associated mammals and birds as simply grassland-associated predators (83%) for further analysis. The three primary grassland-associated predators were striped skunk (48%), thirteen-lined ground squirrel (24%), and rodents (16%). The woodland-associated mammals were white-tailed deer, raccoon, and Virginia opossum (*Didelphis virginiana*); all occurred with equal proportions. Milk snake (*Lampropeltis triangulum*) was the only snake species recorded depredating nests on the control sites.

There were 72 nest predation events recorded on removal sites (including the pre-removal season); of these, 46% were grassland-associated mammals, 39% were snakes, and 15% were woodland-associated mammals. The three primary grassland-associated predators were thirteen-lined ground squirrel (48%), striped skunk (21%), and rodents (21%). The woodland-associated mammals were white-tailed deer (46%), raccoon (36%), and opossum (18%). Fox snakes (89%) were the primary snake predating nests on the removal sites.

**Figure 4:**
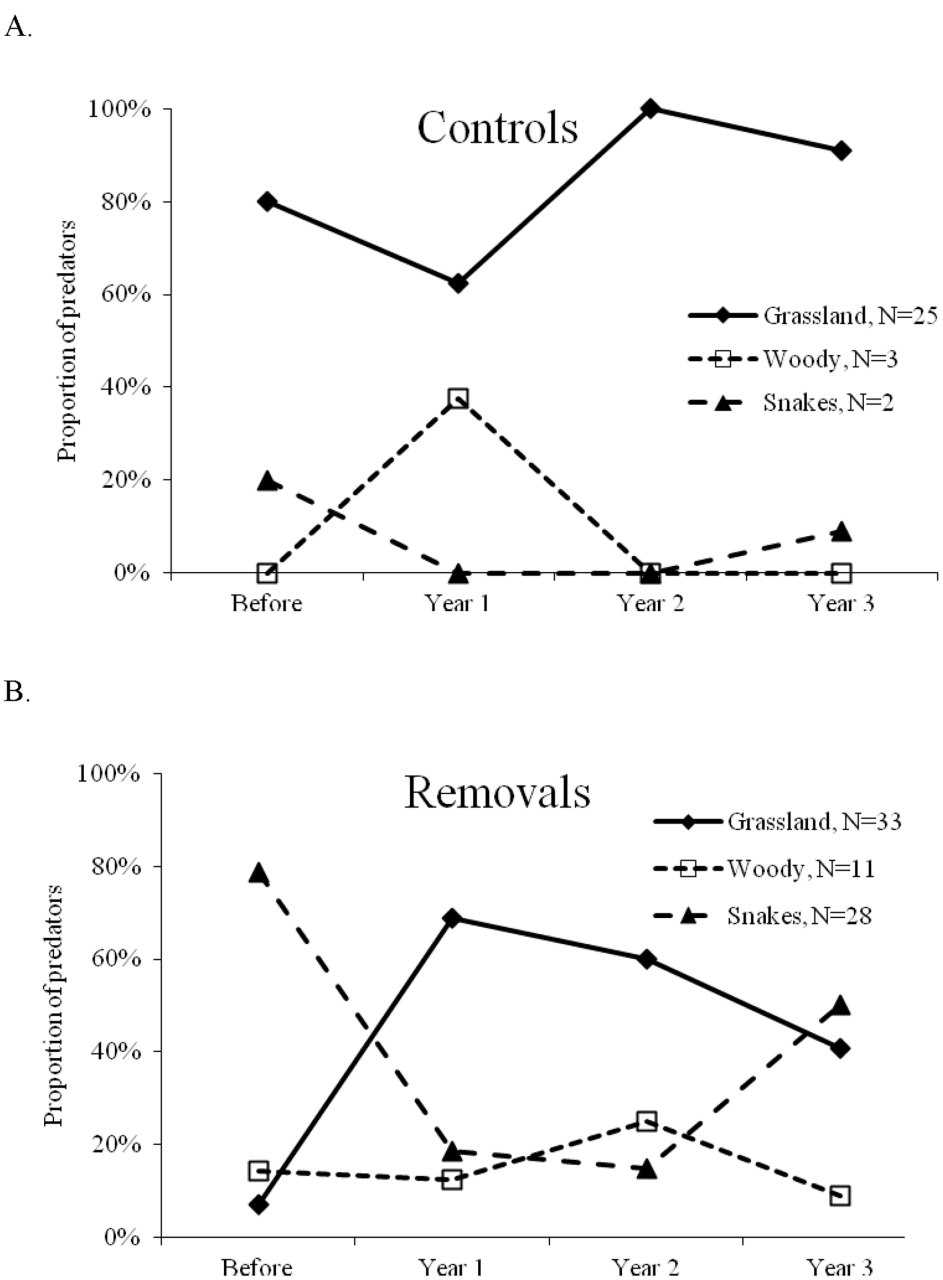
Nest depredation by predator group and year. Proportions for the (A) control and (B) tree row removal sites.

The pattern in the proportions of nest predators across time differed between the control and removal sites (Fig. 4; χ^ 2^ = 24.9, P = 0.008). The majority of nest predation at the control sites was by grassland-based species; there was no change in the proportion of grassland-based nest predators across years on the control sites (χ^2^ = 3.3, P = 0.34). In contrast, following vegetation removal, the proportion of nest predation by snakes declined significantly the first year after tree removal, while the proportion of predation by grassland-based predators increased (χ^ 2^ = 20.2, P = 0.003; Fig. 4). The primary predator contributing to the increase was the thirteen-lined ground squirrel (6 of the 11 grassland-based predators the year after removal).

### Nest Predator Activity

Small rodents and raccoons had the highest activity in the sand track stations at control and pre-removal sites, respectively (Tble 6). The first year following removals, small rodent activity increased fivefold (Table 6). The second and third years following tree row removal, thirteen-lined ground squirrels had the highest activity (Table 6). Thirteen-lined ground-squirrel activity on the removal sites was not correlated with activity on control sites in either edge or interior habitats (covariate term, P>0.25, both tests). On the control sites and pre-removal, this species had lower activity in sand track stations at edges; after removal, activity in the stations at edges increased on the treatment sites 9 to 27 times compared to control and pre-removal levels (Table 6) (quadratic time trend model F = 29.16, df = 2,17, P<0.001). In field interiors, ground squirrel activity in sand track stations also increased after tree removal (linear time trend model F = 4.53, df = 1, 18, P = 0.047); activity at interior stations increased 2 to 3 times the control and pre-removal levels (Table 6).

**Table 6:** Mean percent activity (± SE) for species recorded as common potential nest predators (occurrence/station/day x 100) in tree row edge and grassland interior habitats on the control and tree row removal sites.

	Grassland-associated mammals				Woodland-associated mammals		Snakes
	13-lined ground squirrel	Coyote	Striped skunk	Small rodents	White-tailed deer	Raccoon	Snake spp.
Control:							
Edge	2.8 (1.1)	0.9 (0.4)	1.7 (0.7)	12.3 (3.1)	4.8 (1.5)	11.2 (4.1)	0.4 (0.2)
Interior	8.9 (3.2)	1.6 (0.4)	0.6 (0.2)	11.4 (4.9)	2.9 (0.8)	1.0 (0.3)	0.3 (0.1)
Pre-removal:							
Edge	0.9 (0.5)	0.4 (0.2)	2.2 (0.6)	2.7 (1.2)	2.6 (0.8)	11.7 (6.2)	0.7 (0.7)
Interior	7.9 (5.8)	3.0 (2.1)	3.5 (2.4)	1.7 (1.5)	10.6 (4.4)	1.7 (1.3)	0.9 (0.5)
Year 1:							
Edge	14.2 (5.2)	0.4 (0.2)	0.3 (0.3)	15.7 (7.0)	5.3 (0.7)	1.3 (0.9)	0.5 (0.1)
Interior	10.5 (4.5)	0.8 (0.1)	1.4 (1.1)	17.0 (9.9)	2.9 (1.3)	0	0.9 (0.1)
Year 2:							
Edge	24.7 (3.8)	0.7 (0.3)	0.6 (0.3)	24.0 (9.5)	2.1 (1.4)	1.6 (1.4)	0.5 (0.2)
Interior	22.6 (1.2)	0.8 (0.2)	0.9 (0.9)	15.6 (7.8)	2.6 (1.1)	1.9 (1.3)	0.6 (0.3)
Year 3:							
Edge	16.2 (2.6)	1.3 (0.5)	0.6 (0.6)	5.3 (1.7)	3.1 (0.8)	0.7 (0.7)	0
Interior	15.2 (1.2)	2.9 (1.8)	0	3.6 (0.3)	1.5 (0.8)	1.3 (0.2)	0

Of the other grassland predators, only striped skunk activity in sand track stations changed after tree removal. Specifically, skunk activity at interior stations was lower than activity in stations on the control sites and decreased after removal (linear time trend: F = 5.10, df = 1, 17, P = 0.037) (Table 6). Coyote activity in sand tack stations did not change after removal (linear time trend models, P>0.50, at both edges and interiors) (Table 6) and rodent activity in the stations fluctuated with activity on the control sites (covariate term, P<0.05, at edges and interiors).

Of the woodland-associated nest predators, raccoon activity was highest at edge track stations on the control and pre-removal sites (Table 6). Raccoon activity in stations on the removal sites was not correlated with activity rates in edges of control sites (covariate term, P>0.25). Raccoon activity in edge stations declined after the tree row was removed (linear time trend model F = 4.49, df = 1, 18, P = 0.048) (Table 6). At removals, raccoon activity in interior sand track stations, very low before tree row removal, was correlated with interior raccoon activity on control sites (covariate: F = 4.16, df = 1, 6, P = 0.074) and did not change after removal (linear time trend: F = 1.56, df = 1,6, P = 0.26) (Table 6). White-tailed deer activity in stations on the removal sites was not correlated with activity rates on control sites in either edges or interiors (covariate term, P>0.50, both tests). White-tailed deer activity in interior sand track stations was higher on the removal sites prior to treatment compared to the control sites (F = 7.15, df = 1, 17, P = 0.016) and decreased in interior stations after tree row removal (linear time trend: F = 8.02, df = 1, 17, P = 0.011) (Table 6).

Snake activity in sand track stations was relatively low on all sites (Table 6). In addition, activity on treatment sites was not correlated with activity on the control sites (covariate term, P>0.50, both tests). Snake activity in interior stations was greater on the removal sites prior to treatment compared to the control sites (F = 5.78, df = 1, 17, P = 0.028) and tended to decrease at interiors after tree row removal (linear time trend: F = 4.08, df = 1, 17, P = 0.059).

From the cover board surveys, the Eastern milk snake was the most commonly captured species in 2006, 2007 and 2008 (n = 59, 77, 39) and brown snake (*Storeria dekayi*) was the most commonly captured snake species in 2005 (n = 49). Of the 813 snakes captured, 352 (43%) were considered large enough to depredate nests, these included common garter (*Thamnophis sirtalis*), Eastern plains garter (*T. radix*), Eastern milk, and Western fox snakes (*Elaphe vilpina*).

## Discussion

The removal of tree rows and woody vegetation was an effective means of managing patches of grassland bird habitat in southern Wisconsin. Historically, these areas consisted of grasslands that were often greater in area and not dissected extensively by features such as linear tree rows, and the composition of the predator community was likely different (see [33], where only one of 79 video-recorded nests in large grasslands was depredated by a woodland-based predator). Thus, the removal of focal tree rows generally benefitted our three grassland bird study species by increasing the production of young (Table 7), with caveats explained below.

**Table 7:** Summary of the relative impacts on birds of tree row removal.

	Male density		Nest density		Nests fledged
	Edge	Interior	Edge	Interior	Total site
	F; df^1^; P	F; df^1^; P	F; df^1^; P	F; df^1^; P	Difference^2^
Bobolink	13.5; 0.002**	2.3; 0.15	14.3; 2,6; 0.005**	1.1; 0.29	+10 (20%)
Eastern meadowlark	0.04; 0.83	0.8; 1,17; P = 0.39	3.2; 5,14; 0.039*	1.4; 1,17; P = 0.25	–4 (7%)
Henslow’s sparrow	5.2; 0.035*	0.5; 0.49	7.2; 0.015*	3.2; 0.09	+9 (69%)
Summary	2 of 3 species increased at edges		All species increased at edges		Greater for 2 of 3 species

Comparisons were between data for control sites and pre-removal years versus removal years. Nests fledged were compared for whole sites (Edge+Interior).

1 Degrees of freedom (df) = 1, 18 unless otherwise noted. ^2^Difference of nests fledged after removal minus pre-removal.

Following the removal of tree rows, potential predators were redistributed such that the predator community more closely resembled that associated with larger intact grassland patches (see [33]). While the activity of woodland predators nearly ceased, the activity of grassland predators increased. Also, where present prior to treatment, snakes remained frequent predators. For these reasons, nest survival did not increase consistently among species or seasons. Our overall conclusion is that the removal of tree rows is beneficial, however, the benefits will be affected by the responses of the common nest predator species. Therefore, we recommend that prior to the removal of tree rows, managers consider the potential nest predator community to appropriately scale expectations and prioritize woody vegetation removal. The necessity to formally characterize the predator community at a site would be gauged relative to project scale and costs, as well as the types of predators observed (woodland, grassland, ground squirrels, etc.).

Our results were analogous to those with other forms of grassland management, for example at reclaimed mines [62] and CRP plantings [63], [64], where grassland birds have responded, using previously unoccupied habitat. The removal of tree rows enlarged grassland habitat patches, effectively changing each focal tree row into the core of a new (larger) grassland patch. The cost of the removals was moderate and overall the benefits for grassland birds we documented outweighed the costs. Henslow’s sparrow, the most sensitive of the study species, in terms of habitat and area required [65], [66], as well as the highest priority for grassland bird conservation in eastern and midwestern North America [65], [67], [68] did not occur near tree rows, yet increased in male density and number of nests at all sites where removals took place. Considering edge habitat, <50 m from trees [17], [18], the density of males and nests increased for each species after removal. The sole exception was the male density for Eastern meadowlark. However, this may reflect polygyny, as up to 38–80% of males have more than one mate [69]. Future studies can assess whether female grassland birds select mates on basis of territory placement relative to habitat edges and interiors.

Our finding that nest survival did not improve, contrary to theory, was a result of the changes in the nest predator community. In fact, the predator community shifted; woodland-associated predator (raccoon and striped skunk) activity decreased while grassland-associated predator (thirteen-lined ground squirrel) activity and nest predation dramatically increased at edges where tree rows were removed. Our video identification of nest predators also confirmed this change in nest predators. The response of thirteen-lined ground squirrels to tree removal in our study appears to support the idea that an inverse relationship between distance to habitat edge and nest success for grassland and shrubland birds may be due to the greater abundance of thirteen-lined ground squirrels in patch interiors [25], [70]. However, we note that following tree row removals, ground squirrel activity at edges exceeded that measured in the interiors of pre-treatment and control fields. Therefore, we suggest that thirteen-lined ground squirrels responded to elements of the removals beyond just vegetative structure, e.g., vegetation flush and newly exposed seed bank, areas of disturbed, exposed soil, etc., and that the dramatic difference between former edge and the interior will decrease over time. It is also possible that the smaller grassland predators (snakes and ground squirrels) may exhibit compensatory nest predation, as has occurred elsewhere following the removal of medium-sized mammals [71].

Snake activity remained similar yet predation by snakes decreased by nearly four-fold the two seasons following vegetation removal before increasing to about 50% pre-removal proportion of depredations. The differences between snake activity and nest predation may reflect the availability of alternate prey; for our small sample, rodent activity trended negatively with the proportion of bird nests depredated by snakes (r_s_ = −0.89, P = 0.11).

The inter-relationships among species could also be a factor as we were unable to control for wider-ranging effects of larger cycles such as mast production, predators, parasites, primary consumers (like deer and cattle), etc., that can greatly affect populations of small rodents and, in turn, songbirds [72], [73], [74], [75], [76]. For instance, rodent activity at removals fluctuated closely with that at our controls. However, our treatment may have affected the dynamics of any such cycle(s) as we dramatically altered the activity of predators and competitors: removals decreased two mesopredators (skunk and raccoon), and increased thirteen-lined ground squirrel (for all 3 years post-treatment) and white-tailed deer (for one season). Likewise, trophic interactions operating across multiple scales (see [77]) may take time to coalesce. Future studies could include periodic surveys beyond 3 years post-treatment to determine if and how these communities re-stabilize. For instance, activity of the American badger (*Taxidea taxus*), a specialist predator of ground squirrels, may have increased, particularly outside our survey periods during ground squirrel hibernation (September-May), and this would require more than three seasons after removals to detect.

### Application

Woody vegetation removal would help create habitat for area-sensitive grassland birds in the Midwest such as bobolink, grasshopper sparrow, and, in the Great Plains, Sprague’s pipit (*Anthus spragueii*) (review [66]). Indeed, state and federal agencies recommend practices to curtail planting tree rows and reduce woody vegetation for the benefit of Sprague’s pipit [78], [79]. However, site variability is an important consideration when developing management plans [37]. The nest predators we identified varied among sites, suggesting that understanding site context and predator community composition will be important. For instance, we recorded most predation by snakes at one removal site, where the most garter snakes and the only fox snakes were captured. Thus, pre-existing conditions (e.g., nearby snake hibernacula) and the presence of species not greatly affected by tree row vegetation (e.g., snakes) may impact the effectiveness of habitat management for grassland birds.

Finally, our study has implications for grassland management by landowners, conservation organizations, and technical assistance provided by agencies. In particular, the U.S. Department of Agriculture’s (USDA) CRP (although primarily focused at soil and water conservation) which has helped mitigate the impacts of habitat loss for several grassland species [63], [64], [80], [81] can be improved to further enhance the benefits of such lands for grassland wildlife where it is ecologically appropriate. For example, the recently instituted USDA State Acres for Wildlife Enhancement (SAFE) program aims to target and aggregate CRP acreage in specific areas where it will have the most benefit for focal wildlife species; in many states this includes grassland birds. Guidelines for the CRP and other federal and state agricultural conservation programs, may be designed so that the planting of trees is not incentivized or encouraged in or adjacent to upland grassland habitats, especially those in open grassland landscapes of high conservation priority (see [40]). To be clear, this includes windbreaks, shelterbelts, evergreen plantations and tree groves for fruit and/or lumber production that fragment previously contiguous grassland blocks.

Globally, planning for tree planting in historically tree-less areas may include assessment of ecosystem impacts and longterm outcomes. If woody vegetation is deemed necessary, the focus might be on non-linear configurations (see [40]). Consideration can be made for controlling the long term encroachment and spread of woody vegetation; tree plantings could be concentrated in locations where woodlands predominate on the landscape rather than open grasslands [82].

## Conclusions

The positive impact of the removal of tree rows and associated woody vegetation on male density, nest density, and nest success for the bobolink and Henslow’s sparrow, as well as nest density for the Eastern meadowlark, suggests that tree row removal may be an important practice to manage for these declining species of concern [18], [63], [64], [80], [81], [83]. Changes in the predator community following the removal of tree rows included an unexpected increase in the activity of and nest predation by thirteen-lined ground squirrels. Further studies are required to determine whether thirteen-lined ground squirrels will decrease in association with a reduction in differences in vegetation near and far from the removal areas and/or an increase in ground squirrel predators such as badgers. Under such a scenario, the benefits of tree row removal would be greatly increased through additional gains in nesting productivity.

## Supporting Information

Figure S1 **Schematic maps of sampling design applied at each control site.** Each site is shown with sampling areas outlined (left) and delineations for analysis of grassland/tree row edge versus grassland interior areas (right).
(TIFF)

Figure S2 **Schematic maps of sampling design and tree row removal at each treatment site.** Each site is shown with sampling areas outlined (left) and delineations for analysis of grassland/tree row edge versus grassland interior areas (right). Areas of woody vegetation removal beyond focal tree rows are outlined in green (right).
(TIFF)

File S1 **Supporting information.**
(DOCX)
