# Supplementary figures and images for: Impacts of Tree Rows on Grassland Birds and Potential Nest Predators: A Removal Experiment
Source: PLoS One. 2013 Apr 2;8(4):e59151. doi: 10.1371/journal.pone.0059151 (PMC3614982; doi:10.1371/journal.pone.0059151)

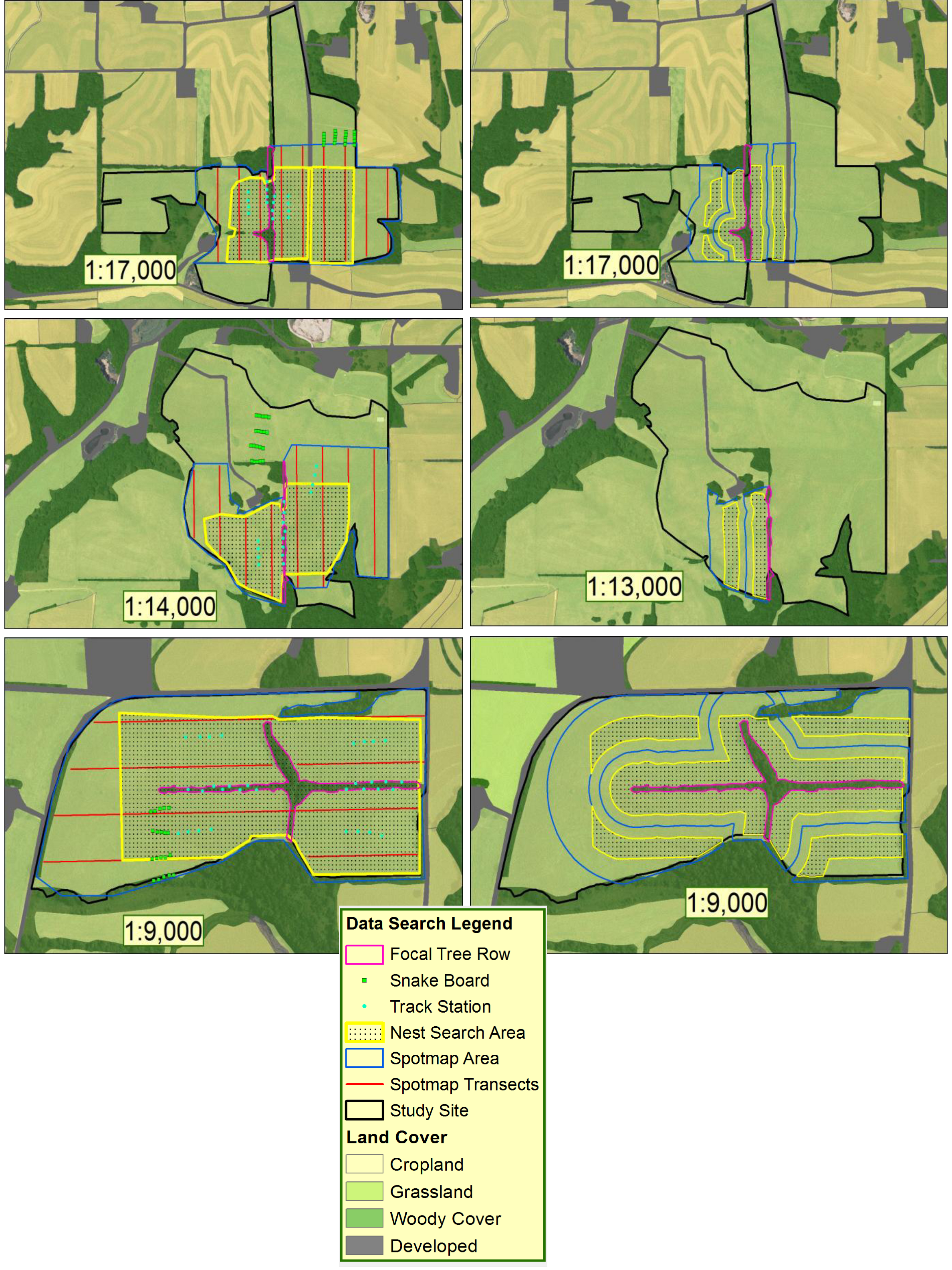

Supplement: Figure S1 — Schematic maps of sampling design applied at each control site. Each site is shown with sampling areas outlined (left) and delineations for analysis of grassland/tree row edge versus grassland interior areas (right). (TIFF) [file pone.0059151.s001.tiff]

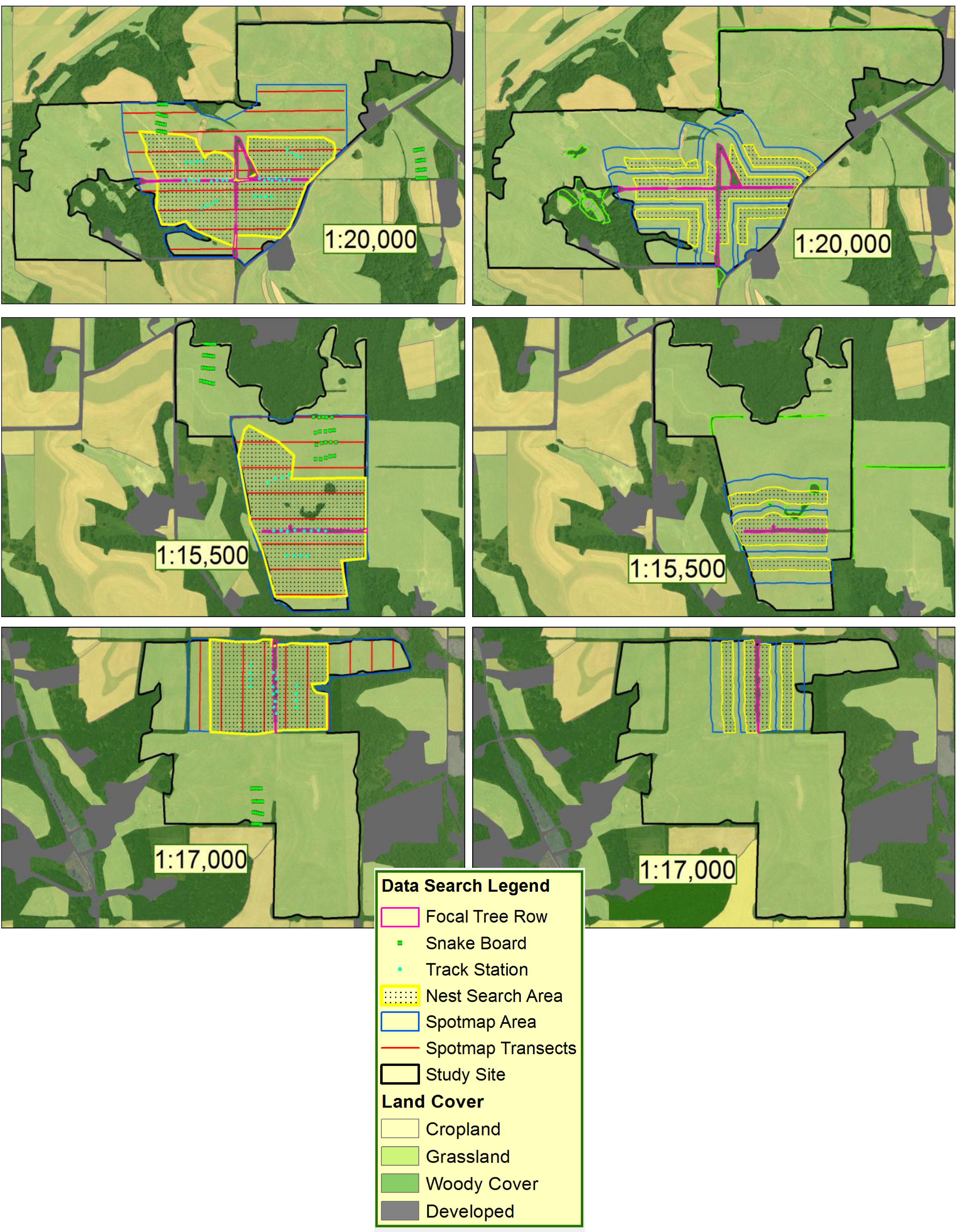

Supplement: Figure S2 — Schematic maps of sampling design and tree row removal at each treatment site. Each site is shown with sampling areas outlined (left) and delineations for analysis of grassland/tree row edge versus grassland interior areas (right). Areas of woody vegetation removal beyond focal tree rows are outlined in green (right). (TIFF) [file pone.0059151.s002.tiff]
