# Supplementary material for: Impacts of Tree Rows on Grassland Birds and Potential Nest Predators: A Removal Experiment
Source: PLoS One. 2013 Apr 2;8(4):e59151. doi: 10.1371/journal.pone.0059151 (PMC3614982; doi:10.1371/journal.pone.0059151)
Supplement: File S1 — Supporting information. (DOCX) [file pone.0059151.s003.docx]

**Supporting Information**

**Supplementary Methods, Results and Discussion**

Methods

*Avian abundance*-

To record bird locations in the field we created 50 m^2^ GPS grid maps plotted over aerial photos of each study site (scale 1:2700) using ArcGIS [1]. With the grid maps and a handheld GPS unit, surveyors could map bird locations to within +/- 25 m. At our largest site, two observers worked simultaneously on either side of the tree row, alternating sides between survey dates (see Figs. S1 and S2).

We digitized bird locations (from first observation of an individual) from each survey round as color-coded point data in ArcGIS [1], and created separate maps, by species, for each year by overlaying all survey rounds together. We then drew convex polygons, enclosing clusters of three or more points considered to be associated with the territory held by an individual male [2]. We used the centroid of the polygon to estimate the proximity of singing male territories to the focal tree row.

*Avian productivity-*

We located most nests by flushing adults from nests while systematically walking abreast (4-10 people spaced at 2 m) through fields, dragging 10-20 m ropes, or sweeping 1.5-3 m poles through the grass when conditions and vegetation structure were appropriate. We also located nests through behavioral observations of nesting activity [3] with and without blinds, and fortuitously while conducting other activities. For the walking and rope-dragging methods, we used 1-m tall wired vinyl flags (in place only momentarily) to keep track of the edges of searched areas and to minimize overlap. Each nest was marked with a wired flag (130 cm^2^ vinyl) placed 4 m north or south of the nest. The flags allowed us to avoid disturbing nests during field activities and to check nests without approaching within 1 m.

When visually checking nests, care was taken not to disturb vegetation near the nest, and to walk past the nest to avoid creating a track or scent trail to the nest. Nest visits rarely lasted greater than 5 s and most could be viewed from > 1 m from the nest. After two consecutive visits without adults, we felt the eggs to determine if they were cool and the nest was abandoned. For nests video-recorded, we checked video to determine if nocturnal nest attendance/incubation had occurred. At empty nests, we used cues such as adult alarm calls, adults carrying food, fledglings near nest, or presence of fresh feces and feather scales along with nestling age to determine whether nests were successful.

*Identification of nest predators-*

We used two types of camera systems during the course of the study. In 2005 and 2006 we used analog videotape recorders (VHS at 2–6 frames/s) and during 2006 through 2008 we used digital video recorders (Archos AV500 at 30 frames/s). Both recording systems were attached to cameras with 25-m cables (following the protocol established by [4]). Each camera was mounted on a wooden dowel 3 - 38 cm above the ground. Cameras were 64 cm^3^ and placed 12 - 25 cm from a selected nest, depending on the nest structure and surrounding vegetation. The field of view at these distances ranged from 414 to 1,320 cm^2^. Cameras were typically placed at or below the height of surrounding vegetation, to avoid creating a visual cue for potential predators. We buried the cable beneath grass litter and camouflaged cameras with nearby grass; this process typically took 10 - 15 min. For VHS recorders, tapes were replaced and nest contents viewed on a monitor every 24 h; batteries were replaced every 24 - 48 h. For digital video recorders, batteries and recorders were replaced and nest contents viewed on the recorder every 48 h.

To identify nest predators and assess nest fates, we reviewed video at nests that were apparently depredated (contents were missing sooner than expected). Since we could not identify predators as individuals, predatory visits by the same species separated by more than an hour were recorded as separate predation events.

*Activity of potential nest predators-*

Sand track stations were placed in 4 sets of 4 stations, with 2 interior sets and 2 tree row sets on each side of the focal tree row (Figs. S1 and S2). Each station was a 1-m^2^ circle of fine sand (~18 L) mixed with 250 ml of mineral oil (see [5]). Mineral oil was used to improve track clarity and was selected because it was non-toxic and odorless so it would not attract animals to the station and create potential bias. All vegetation in the 1-m^2^ circle was clipped prior to depositing sand to ensure improved track registry. Stations within a set were placed 30 m apart from each other. Interior station sets were placed >100m from the tree row. A white, unscented Plaster of Paris disk (Pocatello Supply Depot, Pocatello, ID) was placed in the center of the track station as a novel, visual stimulus to promote track registration. Scented lures were not utilized because they could modify natural movement patterns (drawing species into the station) and we were interested in natural activity rates of animals already moving through the area.

All vegetation in the 1-m^2^ circle was clipped prior to depositing sand to ensure improved track registry. We used field guides [6], [7] to identify species; if there was a question about a track, measurements were taken along with a digital photo for identification by Wisconsin Department of Natural Resources researchers. Stations were reset by smoothing the sand with a small plastic rake and removing any vegetation in the station that could impede track registry. We made efforts to reduce registry bias, including varying the order in which stations were checked, minimizing human scent near the stations (i.e., we wore knee-high rubber boots and minimized contact with vegetation), and varying the walking route to and between stations.

The stations were checked every other day. All tracks in the station were identified to species and recorded. Since tracks are unreliable for distinguishing individuals, we considered each station independently [8] and counted multiple registries by the same species as a single hit for that species. Thus, each track or set of tracks left by a species was recorded as a hit/station/day. After recording all tracks in a station, stations were reset by smoothing the sand with a small plastic rake and removing any vegetation in the station that could impede track registry. Stations disrupted by rain were cleared, but no data was recorded since track identification was unreliable.

*Herbicide application*

After woody vegetation was cut, the tops of remnant stumps were treated with glyphosate to deter re-growth. In July-August following removal, we sprayed Amine 2 2,4-D broadleaf herbicide (active ingredient Dimethylamine Salt of 2-4-Dichlorophenoxyacetic Acid) and Garlon 4 (active ingredient triclopyr) at the recommended dilutions from a quad with a mounted tank and sprayer to promote grass re-colonization and abate regeneration of woody vegetation.

REFERENCES

1. ESRI (2008) ArcGIS version 9.3. Redlands, CA: Environmental Systems Research Institute, 1992-2008.

2. International Bird Census Committee (IBCC) (1970) Recommendations for an international

standard for a mapping method in bird census work. Stockholm: Swedish Nat. Res. Council.

Bull Ecol Research Committee 9:49-52.

3. Winter M, Hawks S, Shaffer J, Johnson DH (2003) Guidelines for finding nests of passerine

birds in tallgrass prairie. Prairie Nat 35:197-211.

4. Renfrew RB, Ribic CA (2003) Grassland passerine nest predators near pasture edges

identified on videotape. Auk 120:371-383.

5. Ribic CA, Guzy MJ, Anderson TA, Sample DW, Nack JL (2012) Bird productivity and nest predation in agricultural grasslands In: Ribic CA, Thompson FR III, Pietz PJ, editors. Video Surveillance of Nesting Birds. Studies in Avian Biology 43, Berkeley: Univ California Press. pp. 119-134.

6. Elbroch M (2003) Mammal tracks and sign, a guide to North American species. Mechanicsburg, PA: Stackpole Books.

7. Murie OJ, Elbroch M (2005) A field guide to animal tracks, third edition. New York: Houghton Mifflin Company.

8. Heske EJ, Robinson SK, Brawn JD (1999) Predator activity and predation on songbird nests

on forest-field edges in east-central Illinois. Landsc Ecol 14:345-354.
